# Effects of Slow Freezing and Vitrification of Human Semen on Post-Thaw Semen Quality and miRNA Expression

**DOI:** 10.3390/ijms25084157

**Published:** 2024-04-09

**Authors:** Rebeka Podgrajsek, Luka Bolha, Tjasa Pungert, Joze Pizem, Katerina Jazbec, Elvira Malicev, Martin Stimpfel

**Affiliations:** 1 Department of Human Reproduction, Division of Obstetrics and Gynaecology, University Medical Centre Ljubljana, 1000 Ljubljana, Slovenia; rebeka.podgrajsek@kclj.si (R.P.);; 2 Institute of Pathology, Faculty of Medicine, University of Ljubljana, 1000 Ljubljana, Slovenia; luka.bolha@mf.uni-lj.si (L.B.); joze.pizem@mf.uni-lj.si (J.P.); 3 Blood Transfusion Centre of Slovenia, Slajmerjeva 6, 1000 Ljubljana, Slovenia; katerina.jazbec@ztm.si (K.J.); elvira.malicev@ztm.si (E.M.); 4 Biotechnical Faculty, University of Ljubljana, Jamnikarjeva ulica 101, 1000 Ljubljana, Slovenia; 5 Faculty of Medicine, University of Ljubljana, 1000 Ljubljana, Slovenia

**Keywords:** semen, cryopreservation, vitrification, slow freezing, spermatozoa, microRNA, assisted reproduction, infertility

## Abstract

Semen cryopreservation has played an important role in medically assisted reproduction for decades. In addition to preserving male fertility, it is sometimes used for overcoming logistical issues. Despite its proven clinical usability and safety, there is a lack of knowledge of how it affects spermatozoa at the molecular level, especially in terms of non-coding RNAs. Therefore, we conducted this study, where we compared slow freezing and vitrification of good- and poor-quality human semen samples by analyzing conventional sperm quality parameters, performing functional tests and analyzing the expression of miRNAs. The results revealed that cryopreservation of normozoospermic samples does not alter the maturity of spermatozoa (protamine staining, hyaluronan binding), although cryopreservation can increase sperm DNA fragmentation and lower motility. On a molecular level, we revealed that in both types of cryopreservation, miRNAs from spermatozoa are significantly overexpressed compared to those in the native semen of normozoospermic patients, but in oligozoospermic samples, this effect is observed only after vitrification. Moreover, we show that expression of selected miRNAs is mostly overexpressed in native oligozoospermic samples compared to normozoospermic samples. Conversely, when vitrified normozoospermic and oligozoospermic samples were compared, we determined that only miR-99b-5p was significantly overexpressed in oligozoospermic sperm samples, and when comparing slow freezing, only miR-15b-5p and miR-34b-3p were significantly under-expressed in oligozoospermic sperm samples. Therefore, our results imply that cryopreservation of normozoospermic sperm samples can modulate miRNA expression profiles in spermatozoa to become comparable to those in oligozoospermic samples.

## 1. Introduction

The cryopreservation of semen is important for fertility preservation in men with infertility issues, men undergoing vasectomy, gonadotoxic therapy, and gender reassignment [1]. The cryopreservation of sperm is not a new discovery, as the first human births from cryopreserved sperm were reported in 1954 [2]. Since then, its clinical use has greatly increased. In 2020, as many as 11,571 ejaculated sperm samples were cryopreserved from a total of 14 European countries [3]. During that time, improvements in cryopreservation have been made, such as the use of new cryoprotectants, freezing carriers, and protocols [4]. Currently, there are two major cryopreservation techniques used in medically assisted reproduction (MAR): conventional slow-freezing and vitrification [5]. Vitrification has recently become popular in clinical practice, especially for the cryopreservation of embryos and oocytes, due to its faster, easier procedure and lower costs [6], but in some cases (e.g., semen cryopreservation), slow freezing is still routinely used.

Regardless of the cryopreservation technique, the cryopreservation of sperm is simple and effective, although it can still have some detrimental impact on sperm at the cellular and molecular levels [7,8]. At the physiological level, cryopreservation has been shown to negatively affect sperm motility [9,10,11,12], vitality [11,12], morphology [9,10,11], and the integrity of the acrosome [11,13] and DNA [9,13,14,15,16,17,18,19]. Studies comparing the impact of slow freezing and vitrification on sperm characteristics have shown conflicting evidence, with some reporting better sperm parameters (motility, viability, and mitochondrial potential) after sperm vitrification [20,21,22,23,24,25,26,27,28,29], while others observing no statistically significant differences [6,30,31,32,33] or better sperm parameters after conventional slow freezing [16,34,35,36,37].

Since the introduction of “omics” technologies, research on the molecular aspects of sperm integrity has shifted toward exploring the impact of cryopreservation through the measurement of RNA molecules, proteins, metabolites, and epigenetic alterations [8]. Currently, the molecular processes affected by sperm cryopreservation remain relatively unknown, especially those associated with epigenetic modifications, which include methylation, histone residue modifications, and modifications by non-coding RNAs (ncRNAs) [38]. ncRNAs are RNA molecules that do not encode protein products and can generally be classified as long ncRNAs (lncRNAs), with a length ≥ 200 nucleotides, or as small ncRNAs (sncRNAs), with a length < 200 nucleotides [18]. A subgroup of sncRNAs are microRNAs (miRNAs), whose regulatory functions in spermatogenesis are related to spermatogonial stem cell renewal and differentiation, the regulation of Leydig and Sertoli cells, the initiation of spermatogenesis during puberty, meiosis, and early embryogenesis [39,40,41,42]. Studies on animal models and humans have shown that miRNA dysregulation can lead to sperm abnormalities and male infertility [43,44,45]. However, the impact of cryopreservation on miRNA expression remains largely unknown, with only a few animal studies [46,47,48,49,50,51,52,53,54] and three studies conducted on humans [46,55,56]. All human studies have been performed using the conventional slow freezing method; therefore, the impact of vitrification on miRNA expression in humans has yet to be defined.

Although whether vitrification is a more appropriate cryopreservation procedure than slow freezing still needs to be elucidated, the objective of our current study was to compare the effects of slow freezing and vitrification on the physiological parameters of spermatozoa, including motility, vitality, and maturity. Additionally, we assessed the effect of semen cryopreservation on the molecular scale by determining the level of DNA fragmentation and the expression of 12 miRNAs, which were selected for analysis based on their previously confirmed role in male infertility. With the obtained results, we confirmed that slow freezing of semen is still superior to semen vitrification in terms of conventional sperm quality parameters and miRNA expression.

## 2. Results

### 2.1. Patient Characteristics

Altogether, 94 normozoospermic patients and 20 oligozoospermic patients were included in our current study. The mean age of the patients in the normozoospermia group was 35.1 ± 5.0 years (mean ± SD). The volume of the semen samples was 3.6 mL (3.0–5.0 mL) (median with interquartile range), the sperm concentration was 57.5 × 10^6^/mL (36.8 × 10^6^/mL–92.0 × 10^6^/mL), the total sperm count was 209.3 × 10^6^ (136.1 × 10^6^–352.7 × 10^6^), and the proportion of normal spermatozoa was 3% (2–5%). In the oligozoospermic group, the mean age of the patients was 36.5 ± 7.7 years, the median volume of the semen sample was 4.7 mL (3.2–5.2 mL), the sperm concentration was 9.5 × 10^6^/mL (7.0 × 10^6^/mL–11.8 × 10^6^/mL), and the total sperm count was 35.8 × 10^6^ (25.5 × 10^6^–51.3 × 10^6^).

### 2.2. Changes in the Sperm Quality Parameters of Normozoospermic Samples after Semen Cryopreservation

To evaluate the effect of the selected cryopreservation methods on spermatozoa, motility and viability were assessed, and different sperm functional tests were also performed. The main results are summarized in Table 1. Overall, we revealed that cryopreservation does not alter the maturity status of spermatozoa as determined by protamine staining or the ability of spermatozoa to bind to hyaluronan as determined by an HBA assay. Furthermore, the proportion of viable spermatozoa was similar between the slow freezing group and the vitrification group (*p* = 0.346). Conversely, we determined that sperm DNA fragmentation significantly increased (*p* = 0.049) after cryopreservation. The post hoc test revealed that this difference was likely due to a significant increase in DNA fragmentation in the semen vitrification group compared to that in the native semen group (*p* = 0.045), while other comparisons did not reveal any significant differences, although this could change if a greater number of samples were analyzed. Significant differences after cryopreservation were also detected for total sperm motility and all assessed types of motility, and in all cases, motility was significantly lower after cryopreservation (*p* < 0.001). Furthermore, when only the slow freezing and vitrification groups were compared, total motility (*p* = 0.007), fast progressive motility (*p* = 0.035), and non-progressive motility (*p* = 0.016) were significantly greater in the slow freezing group, while there was no significant difference in slow progressive motility (*p* = 0.158).

### 2.3. Changes in the Sperm Quality Parameters of Oligozoospermic Samples after Semen Cryopreservation

Due to sample limitations, only motility and viability were assessed in oligozoospermic samples, and in most cases, semen samples were subjected to only one cryopreservation method. The detailed results are presented in Table 2. In general, the quality of native semen samples before slow freezing or vitrification was comparable between the groups (*p* = 0.976 for sperm concentration, *p* = 0.741 for total sperm count, and *p* = 0.889 for sperm total motility). Notably, compared with those of the native samples, the quality of the semen samples after cryopreservation significantly changed, and all types of motility were significantly lower after slow freezing and vitrification (Table 2). However, when motility was compared between spermatozoa subjected to slow freezing or vitrification, no significant difference was detected (*p* = 0.465 for total motility, *p* = 0.936 for fast progressive motility, *p* = 0.091 for slow progressive motility, and *p* = 0.764 for non-progressive motility). Similarly, no difference was observed in sperm viability (15.1% ± 7.7% after slow freezing and 12.4% ± 6.5% after vitrification, *p* = 0.361).

### 2.4. Cryopreservation Alters miRNA Expression in Spermatozoa from Normozoospermic Patients

**Figure 1:**
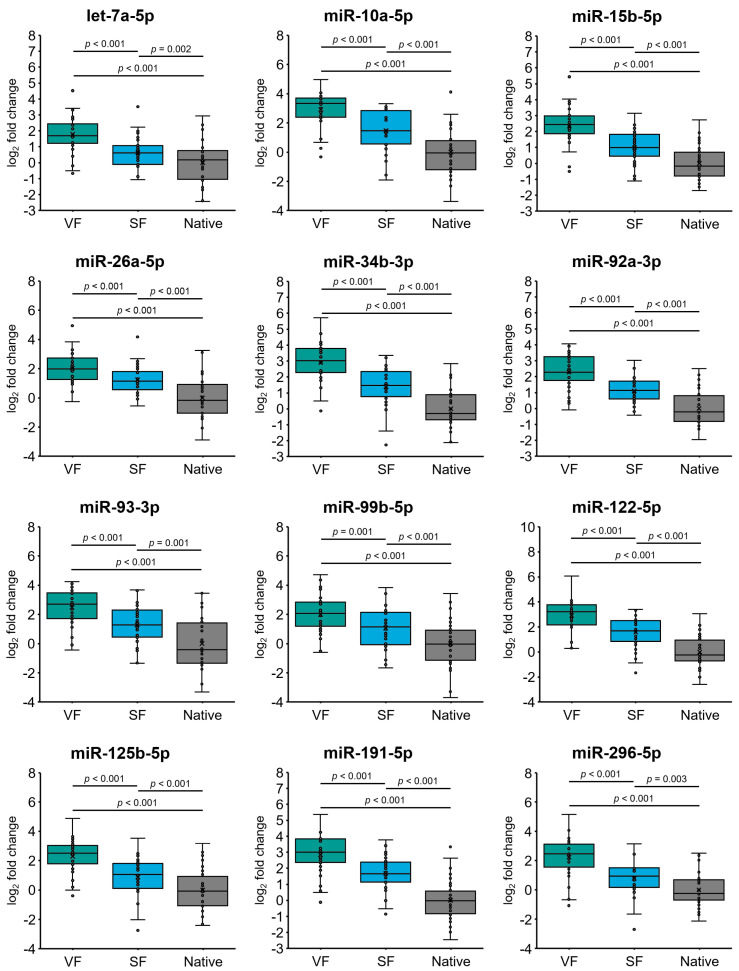
MicroRNA (miRNA) expression in spermatozoa from normozoospermic individuals under different sperm cryopreservation conditions. Expression profiles of 12 selected miRNAs in spermatozoa from 30 individuals, which were partitioned and subjected to vitrification (group VF; n = 30) and to a slow freezing (group SF; n = 30) and compared to native spermatozoa (group Native; n = 30). The horizontal line within the boxplot denotes the median, and the horizontal border lines denote the interquartile range. Each dot represents the log_2_ miRNA fold change of an individual sample. The symbol × denotes the average log_2_-fold change value. The data were evaluated in a pairwise manner using paired Student’s *t* tests. A *p* value of <0.05 was considered to indicate statistical significance.

Under our experimental conditions, we revealed significant 3.5- to 7.6-fold (log_2_-fold change values ranging from 1.8 to 2.9) overexpression of all 12 included miRNAs (all *p* < 0.001) in the spermatozoa from normozoospermic individuals subjected to vitrification (group VF) compared to native spermatozoa (group Native) (Figure 1). In addition, similar expression profiles of the analyzed miRNAs were determined in normozoospermic spermatozoa subjected to slow freezing (group SF) compared to native spermatozoa (group Native), where all miRNAs were overexpressed 1.6- to 3.2-fold (log_2_-fold change values ranging from 0.6 to 1.7), with *p* values ≤ 0.003 (Figure 1). Notably, the expression of all 12 miRNAs significantly differed between the VF and SF groups (all *p* ≤ 0.001), showing overexpression in the VF group compared to the SF group (Figure 1). Overall, our results show that cryopreservation affects miRNA expression in normozoospermic sperm samples and indicate that vitrification has a more pronounced impact on miRNA alteration (i.e., induction) than the slow freezing technique.

### 2.5. Effect of Cryopreservation on miRNA Expression in Oligozoospermic Sperm Samples

**Figure 2:**
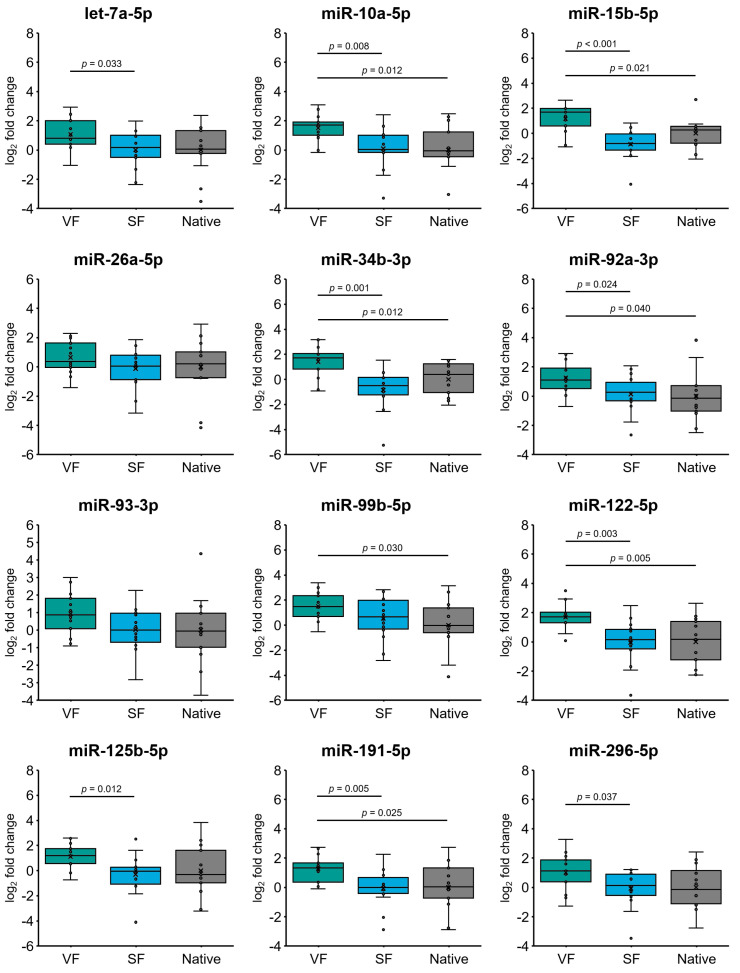
MicroRNA (miRNA) expression in spermatozoa from patients with oligozoospermia under different sperm cryopreservation conditions. Expression profiles of 12 selected miRNAs in spermatozoa subjected to vitrification (group VF; n = 13) and a slow freezing technique (group SF; n = 14) compared to native spermatozoa (group Native; n = 13). The horizontal line within the boxplot denotes the median, and the horizontal border lines denote the interquartile range. Each dot represents the log^2^ miRNA fold change of an individual sample. The symbol × denotes the average log^2^-fold change value. The data were evaluated using the unpaired Student’s *t* test. A *p* value of <0.05 was considered to indicate statistical significance.

When assessing the effect of sperm cryopreservation on miRNA expression in spermatozoa from patients with oligozoospermia, we detected significant 2.2- to 3.2-fold (log_2_-fold change values ranging from 1.2 to 1.7) overexpression of miR-10a-5p/-15b-5p/-34b-3p/-92a-3p/99b-5p/-122-5p/-191-5p (all *p* ≤ 0.04) in the VF group compared to the control group (Native) (Figure 2). Moreover, let-7a-5p and miR-10a-5p/-15b-5p/-34b-3p/-92a-3p/-122-5p/-125b-5p/-191-5p/-296-5p were significantly overexpressed by 1.7- to 4.8-fold (log_2_ fold change values ranging from 0.8 to 2.3) (all *p* ≤ 0.037) in the VF group compared to the SF group (Figure 2). The expression profiles of all included miRNAs did not differ between the SF group and the Native control group (Figure 2), indicating that a slow freezing technique has little effect on miRNA alterations in oligozoospermic sperm samples compared to those in native semen. Similar to sperm samples from normozoospermic individuals, vitrification had a notable impact on miRNA overexpression in spermatozoa from patients with oligozoospermia.

### 2.6. miRNA Expression in Spermatozoa from Patients with Oligozoospermia under Each Sperm Cryopreservation Condition

**Figure 3:**
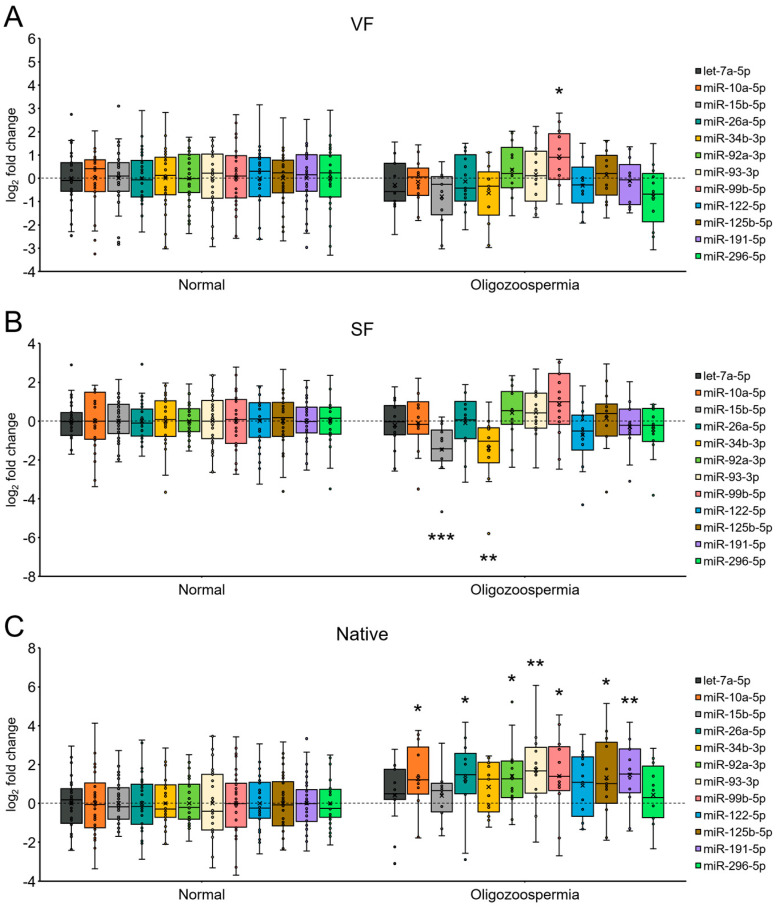
MicroRNA (miRNA) expression in spermatozoa from patients with oligozoospermia compared to normal spermatozoa under each sperm cryopreservation condition. Expression profiles of 12 selected miRNAs in spermatozoa from patients with oligozoospermia compared to spermatozoa from normozoospermic individuals following vitrification (group VF) (**A**) or slow freezing (group SF) (**B**) and in native spermatozoa (group Native) (**C**). Analysis was performed on 13 oligozoospermic and 30 normozoospermic samples in the VF and Native groups (**A**,**C**) and on 14 oligozoospermic and 30 normozoospermic samples in the SF group (**B**). The horizontal line within the boxplot denotes the median, and the horizontal border lines denote the interquartile range. Each dot represents the log_2_ miRNA fold change of an individual sample. The symbol × denotes the average log_2_-fold change value. The data were evaluated using the unpaired Student’s *t* test. For miR-34b-3p and miR-92a-3p, the Mann-Whitney U test was used due to non-normal data distribution. An asterisk indicates a significant difference compared with the control normozoospermic group (* *p* < 0.05; ** *p* < 0.01; *** *p* < 0.001). A *p* value of <0.05 was considered to indicate statistical significance.

We next assessed differences in miRNA expression between oligozoospermic and normozoospermic native semen samples and between semen samples following each cryopreservation procedure (Figure 3). Our analysis revealed that miR-99b-5p was the only significantly differentially expressed miRNA in spermatozoa from patients with oligozoospermia compared to spermatozoa from normozoospermic individuals following vitrification (group VF) and was overexpressed 1.9-fold (log_2_-fold change value 0.9) (*p* = 0.033) (Figure 3A). Conversely, a significant 2.8-fold (*p* < 0.001) and 2.6-fold (*p* = 0.004) under-expression was determined for miR-15b-5p and miR-34b-3p, respectively, and was detected in spermatozoa from patients with oligozoospermia compared to spermatozoa from normozoospermic individuals following the slow freezing technique (group SF) (Figure 3B). In native spermatozoa, of the 12 included miRNAs, miR-10a-5p/-26a-5p/-92a-3p/-93-3p/-99b-5p/-125b-5p/-191-5p were significantly overexpressed 2.4- to 3.3-fold (log_2_-fold change values ranging from 1.3 to 1.7) (all *p* ≤ 0.028) in oligozoospermic samples compared to the control normozoospermic group (Figure 3C). Overall, the results obtained under our experimental conditions imply a possible moderate degree of cryopreservation-induced masking of miRNA alterations in spermatozoa from oligozoospermic patients compared to normozoospermic individuals, which is notable in native spermatozoa but not in spermatozoa that underwent cryopreservation by either vitrification or slow freezing.

## 3. Discussion

In this study, we compared the impact of two different sperm cryopreservation methods (i.e., slow freezing and vitrification) on post-thaw spermatozoa quality and the expression of selected miRNAs. Notably, the study included human semen samples of good and poor quality. Considering the impact on sperm post-thaw quality, our results revealed that cryopreservation of normozoospermic samples does not alter the maturity of spermatozoa, although cryopreservation can increase sperm DNA fragmentation and lower spermatozoa motility. Furthermore, we showed that motility is lower after vitrification than after slow freezing in normozoospermic samples; however, in oligozoospermic samples, we detected no differences. Interestingly, the viability of spermatozoa was similar between the two types of cryopreservation. To the best of our knowledge, this is the first study to evaluate the influence of vitrification on miRNA expression in human spermatozoa, and furthermore, this is the first study to compare slow freezing of semen and semen vitrification in such a manner. Overall, both types of cryopreservation significantly induced the overexpression of the tested miRNAs in cryopreserved semen compared to native semen from patients with normozoospermia, an effect that was observed only after vitrification in the oligozoospermic sample group.

As mentioned, semen cryopreservation has recently become widely used in MAR to combat male infertility due to different health issues, but sometimes it is used only to overcome logistical issues. Regardless of the reason for semen cryopreservation, it is of great interest to all medical workers to determine the best procedure for cryopreservation to obtain the healthiest spermatozoa after thawing. Although semen cryopreservation is generally recognized as safe and efficient, it is known to be challenging and disadvantageous [7]. Cryopreservation causes cryodamage, which can result in impaired motility and viability of spermatozoa, acrosomal changes, and increased sperm DNA fragmentation [11,16,17]. Some of these negative effects were also observed in our current study for both types of cryopreservation procedures. Although slow freezing and vitrification yielded similar results in the oligozoospermic group, it appears that slow freezing has fewer negative effects on post-thaw quality than vitrification in normozoospermic samples. While some studies have shown comparable results in post-thaw sperm quality between slow freezing and vitrification [6,30,31,32,33], other studies have reported results similar to ours. For instance, it has been reported several times that spermatozoa motility is lower after vitrification than after slow freezing [6,34,36,37], with similar viability [35], sperm DNA fragmentation [6,34,35,37], and hyaluronan binding [35]. Contrary to our results, chromatin condensation is also more impaired after vitrification [34]. In contrast to these reports and our data, several studies have shown that spermatozoa motility can be higher after vitrification than after slow freezing [12,20,22,23,25,27,28], with lower sperm DNA fragmentation [20,21,22,23,27], if we mention only two of the most important sperm post-thaw quality factors. Regardless of the cryopreservation approach used, the reason for lower motility after cryopreservation is in impaired function of mitochondria [57,58], where excessive oxidative stress likely damages mitochondrial energy production, due to non-optimal conditions during cryopreservation [59,60,61]. Nonetheless, improvement of these conditions, for instance, with the addition of mitochondria-targeted antioxidants to the cryopreservation medium, the oxidative stress can be reduced, and post-thaw spermatozoa quality markedly improved [58,62].

There are several possible reasons why there have been contradictory results reported in studies comparing slow freezing and vitrification of human semen, including the different qualities of the included native semen samples and the different approaches used for slow freezing and vitrification. The latter may vary in the type of vitrification media and in different types of vials and straws used for vitrifying and storing the vitrified sample. Therefore, to better elucidate the value and clinical potential of vitrification, compared to slow freezing, it is critical to utilize more standardized vitrification protocols, starting with vitrification media whose preparation has not been standardized yet.

Despite the currently widely accepted fact that semen cryopreservation, regardless of the approach used, has some negative effect on post-thaw sperm quality parameters, very little is known about alterations at the molecular level that follow sperm cryopreservation, especially in terms of ncRNAs, particularly miRNAs. The role of miRNA dysregulation in male infertility has been intensively explored in recent years, and miRNAs have been proposed as a new group of putative biomarkers for male fertility and semen quality [63,64]. Despite the proven role of miRNAs in male infertility, data on miRNA dysregulation in spermatozoa induced by semen cryopreservation currently remain scarce. To the best of our knowledge, only three studies have explored this specific topic in humans [46,55,56], and only a few additional studies have been performed in animal models [46,47,48,49,50,51,52,53,54]. Studies using human samples have shown that miRNA expression in spermatozoa significantly changes after sperm cryopreservation. For instance, Ezzati et al. [55] showed that the expression of miR-34c and miR-184 significantly decreased after thawing, while Xu et al. [46] confirmed the downregulation of three miRNAs and the upregulation of 18 miRNAs after thawing. These differentially expressed miRNAs have been shown to be involved in the extrinsic apoptotic signaling pathway, cellular response to DNA damage stimulus, actin cytoskeleton organization, in utero embryonic development, positive regulation of cell migration, and regulation of small GTPase-mediated signal transduction [46]. Furthermore, Huang et al. [56] showed that with increasing cryopreserved semen storage time, the changes in miRNA expression are even more prominent. Nevertheless, from the view of reproduction, it is concerning that most of the identified storage time-dependent differentially expressed miRNAs have mRNA targets expressed in oocytes [56]. In line with these studies, we also observed significant changes in miRNA expression in spermatozoa following cryopreservation in our current study. Interestingly, all 12 of the analyzed miRNAs were overexpressed after cryopreservation in normozoospermic samples after both slow freezing and vitrification. Importantly, all the miRNAs were significantly more overexpressed in spermatozoa subjected to vitrification than in those subjected to the slow freezing protocol. However, in oligozoospermic samples, we observed significant changes in the expression of the tested miRNAs only after vitrification, while the miRNA expression profiles did not differ between native semen samples and samples subjected to a slow freezing protocol. The effect of different cryopreservation procedures on aberrant miRNA expression with regard to semen quality could be most reliably elucidated by comparing miRNA expression profiles between native semen samples, since we revealed that miRNAs are mostly significantly overexpressed in native oligozoospermic samples compared to native normozoospermic samples. Conversely, when vitrified normozoospermic and oligozoospermic samples were compared, we determined that only miR-99b-5p (out of 12 assessed miRNAs) was significantly overexpressed in oligozoospermic sperm samples. Moreover, when comparing sperm samples subjected to slow freezing, only miR-15b-5p and miR-34b-3p (out of 12 assessed miRNAs) were significantly under-expressed in oligozoospermic sperm samples compared to normozoospermic samples. Therefore, our results imply that cryopreservation of normozoospermic sperm samples can modulate miRNA expression profiles in spermatozoa to become comparable to those in oligozoospermic samples.

The expression of 12 included miRNAs in our current study has been assessed previously and their expression profile related with various semen quality parameters and/or male fertility in humans (Table 3). Of these miRNAs, miR-34b-5p, miR-15b-5p, and miR-99b-5p were intensively studied. As such, the expression of miR-34b-3p in spermatozoa was negatively correlated with male age [64] and positively correlated with sperm concentration [65], whereas miR-34b-3p overexpression was even correlated with live births after the ICSI procedure with sperm from patients with teratozoospermia [66]. miR-34b-3p is a member of the miR-34 family, which is crucially involved in regulation of the cell cycle, apoptosis, and senescence [67,68,69]. It has been shown that miR-34b-3p potentially regulates 250 genes [70], and some of the target genes are important regulators of germ cell development, including spermatogenesis, as revealed for NOTCH1, LGR4, VEZT, MAN2A2, and FOXJ2 in testicular tissue of the rhesus monkey [71]. For NOTCH1, Batista et al. [72] showed that it is also dynamically transcribed in preimplantation mouse embryos, and when deregulated, it could affect blastocyst development and hatching. In addition, importance of NOTCH1 in postimplantation embryo development has been elucidated in mice [73], where it has a crucial role in segmentation of somites in the second half of gestation [74]. As revealed, if the NOTCH1 expression was impaired, cell death occurred, and the embryo did not survive [73,74]. Another important miR-34b target is DAZL, which is crucial for mouse germ cell differentiation, since its depletion leads to the absence of gamete production [75]. Besides the testis, miR-34b expression was also detected in ovaries (in *Bos taurus*) although, its expression was not determined in spermatozoa and oocytes [76]. miR-15b-5p is a member of the miR-15/107 family and is downregulated in oligozoospermic patients [64,65]. Expression of miR-15b-5p in trophoblasts has been negatively correlated with the fetus development during early pregnancy [77], and its expression in placental samples was negatively correlated with preeclampsia [78]. According to Wang et al. [79] there are over 400 experimentally validated target genes of miR-15b-5p, and Salas-Huetos et al. [80] further predicted that there are 64 target genes closely related to embryonic morphogenesis and 41 target genes closely related to chromatin modification, which indicates that spermatozoa-derived miR-15b-5p could significantly influence the development of an embryo. miR-99b-5p constitutes the miR-99 family along with miR-99a-5p and miR-100. We have previously shown that miR-99b-5p expression in spermatozoa positively correlates with a good-quality day 3 embryo rate [81]. It was experimentally confirmed in epithelial cells that target genes of this miRNA family comprise mTOR, HOXA1, SMARCA5, CTDSPL, NMT1, CTDSPL, and TMEM30A [82]. Some of these genes play a pivotal role in the development of genetic and malignant diseases and even in early embryonic development. For instance, it was shown for HOXA1 that loss of its function leads to defects in the brainstem, inner ear, cranial ganglia, and in cardiovascular abnormalities [83]. Furthermore, it was shown that its co-expression with PRDM14 is crucial for normal differentiation of epiblast cells to primordial germ cells, although after differentiation its expression in primordial germ cells is absent [84]. In the process of gametogenesis, the expression of mTOR is also crucial. It was shown that its deregulation leads to errors during meiotic and mitotic chromosomal disjunction, it helps to maintain the spermatogonial stem cell pool in the testis and blood–testis barrier, and it regulates premature ovarian follicle loss in ovaries [85]. In addition, it has been shown that mTOR plays an important role in embryo and placenta development [85]. Similar functions have been determined for SMARCA5, whose depletion leads to growth arrest of the embryoblast and the trophectoderm [86] and in failure of the oocyte meiotic resumption [87]. Although the other miRNAs analyzed in this study also showed cryopreservation-dependent differential expression in at least one comparison, they were all previously correlated with male infertility or semen quality in humans and/or in animal models [63,64,65,88,89,90] and not with semen cryopreservation in humans.

Despite the fact that our study is the first to compare the effect of two types of semen cryopreservation procedures on post-thaw sperm quality and miRNA expression employing human semen samples of different qualities, this study has several limitations. The main limitation represents a relatively small number of included miRNAs. Nevertheless, by including a set of miRNAs strongly associated with semen quality, we gained valuable insight into how sperm cryopreservation affects miRNA expression in human spermatozoa and how these altered miRNA profiles reflect sperm post-thaw quality parameters. Overall, further in-depth studies are needed, employing high-throughput techniques (e.g., RNA-Seq), to thoroughly evaluate the influence of semen cryopreservation on spermatozoa miRNA dysregulation and to elucidate how miRNA dysregulation affects cell processes associated with male infertility through interaction with their target genes. Furthermore, other protocols and/or cryopreservation media used in either slow freezing or vitrification should be evaluated to obtain an optimal approach, with as little negative impact as possible on the molecular features of preserved spermatozoa. It would also be most beneficial if larger patient cohorts were enrolled into this and other similar studies, particularly in the oligozoospermic group, preferably in a multicentric setting, to gain a more generalized insight into the role of sperm cryopreservation on spermatozoa biology.

In summary, this is the first study to evaluate miRNA expression in human spermatozoa following sperm cryopreservation, employing slow freezing and vitrification, and the first study to compare miRNA expression profiles between the two cryopreservation methods. Notably, we revealed that cryopreservation in any form results in miRNA alteration compared to native semen. However, semen quality emerged as a crucial factor in contextualizing the effect of cryopreservation on miRNA dysregulation since we determined differential miRNA expression between native normozoospermic and native oligozoospermic semen samples. Therefore, it is a necessity for future studies to consider evaluating additional vitrification approaches to obtain new optimized protocols providing an enhanced post-thaw quality of spermatozoa on both the macroscopic and molecular level. This is of utmost importance, since spermatozoa-derived miRNAs are delivered into oocytes at fertilization, where miRNA dysregulation may influence the fertilization process and the development of the preimplantation embryo. Therefore, potential utilization of novel miRNA biomarkers may crucially contribute to an effective molecular assessment of the post-thaw spermatozoa quality, promoting fertilization rates and successful embryo development.

## 4. Materials and Methods

### 4.1. Study Design

**Figure 4:**
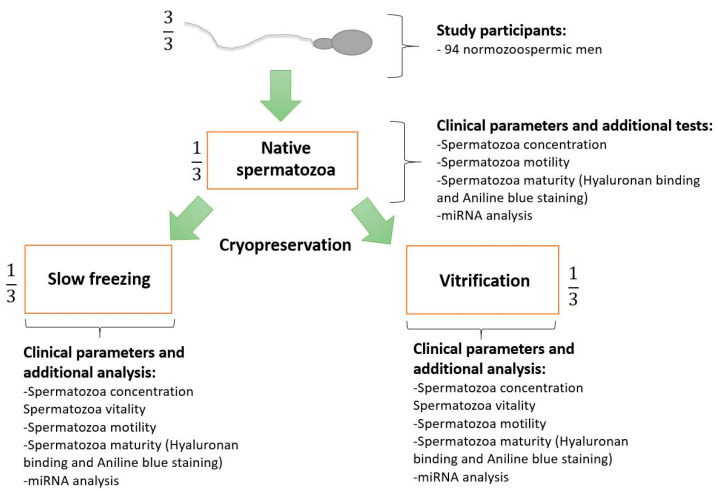
Overview of the study design for normozoospermic patients.

Semen samples from 94 normozoospermic and 20 oligozoospermic men were collected after the diagnostic spermiogram was performed at our andrology laboratory (Department of Human Reproduction, University Medical Centre (UMC) Ljubljana, Slovenia). In this study, we included into the normozoospermic group samples with a concentration of ≥16 × 10^6^ spermatozoa/mL, motility of >42%, a semen volume of ≥2.5 mL and samples without presence of anti-sperm antibodies; however, we neglected morphology, and we did not use it as an inclusion or exclusion factor. In the oligozoospermic group, we included samples with a concentration of <16 × 10^6^ spermatozoa/mL, a semen volume of ≥1.4 mL, and samples without presence of anti-sperm antibodies, while we neglected motility and morphology, and we did not use these parameters as inclusion or exclusion factors. We excluded from our study from both groups semen samples where agglutinations where observed, and semen samples where more than 1 × 10^6^ round cells were observed. Patients invited in our study were not interviewed about their medical history, possible health issues, taking medications or dietary supplements, or about their lifestyle. Patients enrolled between March 2022 and January 2023 were included in the study. In the case of normozoospermic samples, the samples were divided into three equal-volume aliquots. One aliquot of semen was used and stored as a native sample, one was slowly frozen, and one was used for vitrification. After sperm thawing/warming, motility, viability, and hyaluronan binding assays, sperm DNA fragmentation analyses, aniline blue staining of protamines, and miRNA expression analyses were performed (Figure 4). Due to the limited sample volume after performing the spermiogram, not all analyses were conducted for each patient. Nevertheless, the analysis that was performed was conducted for all three conditions (native semen, slow-frozen semen, and vitrified semen). Due to the low sperm count and limited volume of oligozoospermic samples, these samples were collected only for miRNA expression analysis. Because at least 5 × 10^6^ spermatozoa per sample are needed in the RNA isolation procedure to obtain satisfactory yield and purity of isolated total RNA for use in miRNA expression analyses, according to our validation experiments, the samples of each oligozoospermic patient were mostly used for analysis only under one condition (i.e., native sample, slow-frozen sample, or vitrified sample). Notably, each analysis was performed in an equal number of replications.

### 4.2. Study Participants and Semen Collection

The samples included in our current study were collected from semen samples that remained after performing the diagnostic spermiogram and only if written informed consent was obtained from the patients. Spermiograms were performed according to the sixth edition of the WHO manual [105]. Briefly, patients collected semen with masturbation and ejaculation into a sterile collection container after 2–7 days of sexual abstinence. The spermatozoa concentration and motility were assessed with a computer-assisted spermatozoa analysis system (IVOS II., Hamilton Thorne Inc., Beverly, MA, USA). Normozoospermic samples were then divided into three equal aliquots, where one aliquot was analyzed or further processed, while the other two aliquots of native semen samples were cryopreserved (with slow freezing and/or with vitrification).

### 4.3. Semen Cryopreservation

#### 4.3.1. Slow Freezing

In our study, we used the Sperm Freezing Medium (CooperSurgical^®^, Origio, Måløv, Denmark) for slow freezing. Before use, the medium was left to warm to room temperature. The medium was then added by droplets to the semen sample and gently mixed. The volume ratio between the semen sample and Sperm Freezing Medium was 1:1. The mixture was then incubated for 10 min at room temperature and loaded onto high-security sperm straws (CryoBio System, L’Aigle, France). The straws were then placed on liquid nitrogen vapor in a horizontal position for 30 min. After the elapsed time, the straws were transferred to a liquid nitrogen container at a temperature of −196 °C for at least 24 h before thawing.

#### 4.3.2. Vitrification

Because there is no commercially available sperm vitrification medium, we prepared the vitrification medium in-house, which consisted of sperm preparation medium (CooperSurgical^®^) supplemented with 0.5 M sucrose (Sigma-Aldrich^®^, Steinheim, Germany) and 10 mg/mL of human serum albumin (Vitrolife, Västra Frölunda, Sweden). Before cryopreservation, the vitrification medium was left to warm to room temperature. Then, the vitrification medium was added by droplets to the semen sample and gently mixed. The volume ratio between the vitrification medium and semen samples was 1:1. The mixture was then incubated for 5 min at room temperature. In the meantime, a Styrofoam box with liquid nitrogen was prepared. The 25–30 µL droplets were then dropped directly into the liquid nitrogen at an angle of 45° to form solidified droplets. The solidified droplets were then transferred with tweezers into cryovials and stored in liquid nitrogen containers at −196 °C for at least 24 h before warming.

### 4.4. Semen Thawing/Warming

#### 4.4.1. Slow Freezing

The straws with spermatozoa were thawed in a preheated water bath (37 °C). After 5 min of incubation, the content of the straws was transferred to a centrifuge tube and washed with sperm preparation medium warmed to room temperature.

#### 4.4.2. Vitrification

The contents of the cryovials were transferred to 5 mL of preheated (42 °C) sperm preparation medium and incubated until all solidified droplets dissolved. The samples were then mixed and centrifuged at 1400 rpm for 10 min at room temperature to concentrate and wash the samples. Four milliliters of supernatant was discarded, and the pellets were resuspended in the remaining 1 mL of sperm preparation medium.

### 4.5. Determination of Spermatozoa Viability

After thawing/warming, sperm viability was tested using eosin Y/nigrosine staining. Samples from 37 men with normozoospermia and 18 men with oligozoospermia were used for the viability analysis. Briefly, one drop of eosin Y was added to one drop of semen sample and mixed, and after 3 min of incubation, one drop of nigrosine was added to the mixture and mixed. After 3 min, one drop of the mixture was added to a slide to prepare a smear. The samples were then air-dried, and the spermatozoa were assessed under a light microscope at 400× magnification. Viable spermatozoa were unstained, while dead spermatozoa were stained pink. Based on the data, the percentage of viable spermatozoa was calculated.

### 4.6. Sperm Hyaluronan Binding Assay

Sperm Hyaluronan Binding Assay (CooperSurgical^®^) was performed for native samples and samples subjected to slow freezing and vitrification. The analysis was performed on normozoospermic semen samples from 32 patients. Approximately 10 μL of semen was added to the assay chamber and covered with a cover slip. The samples were then incubated for 10 min at room temperature to enable the binding of the mature spermatozoa. Following the incubation period, the number of bound and unbound spermatozoa was counted under a microscope at 400× magnification. Where possible, 200 spermatozoa were counted. The result was then presented as the percentage of hyaluronan-bound spermatozoa, representing mature spermatozoa.

### 4.7. Aniline Blue Staining

The maturity of spermatozoa was also assessed with an additional test based on the determination of maturity of the spermatozoan nucleoprotein. Staining was performed on native, slow-frozen, and vitrified semen samples from 38 normozoospermic men. The samples were stained using the Kit for Determination of the Maturity of Spermatozoa (SpermFunc^®^, BRED Life Science, Shenzhen, China) according to the manufacturer’s protocol. Briefly, after thawing the samples at room temperature (previously stored at −20 °C), multiple washing steps with the addition of 1 mL of normal saline were performed. The samples were centrifuged at 2500 rpm for 5 min at room temperature. After discarding all of the remaining supernatant, 0.1–0.2 mL of solution A was added. The spermatozoa concentration was then adjusted to 20–40 × 10^6^ spermatozoa/mL with the addition of solution A. After adjusting the proper spermatozoa concentration, 5 μL of the sample was applied evenly on the slide and left to dry for 15–20 min. Solutions B, C, D, and E were used for staining according to the manufacturer’s protocol. After applying the last staining solution, the slide was washed with tap water and left to dry at room temperature. After the slides were completely dry, two drops of Ultrakitt mounting medium (J.T.Baker) were added, and the slides were covered with a cover slip and left to dry for 30 min. Then, at least 200 spermatozoa were assessed under a microscope at 400× magnification. The spermatozoa with mature nucleoprotein were stained pinkish red, whereas spermatozoa with immature nucleoprotein were stained cyan. The percentages of spermatozoa with immature and mature nucleoprotein were calculated.

### 4.8. Sperm DNA Fragmentation Analysis (TUNEL Analysis)

For the determination of spermatozoa DNA fragmentation, semen samples from 26 normozoospermic patients were used. Sperm DNA fragmentation was assessed using the APO-DIRECT™ Kit (BD Pharmingen™, San Diego, CA, USA), as instructed by the manufacturer. Briefly, semen samples were washed with 1× PBS buffer (Gibco^®^, Grand Island, NY, USA) (centrifugation at 1400 rpm for 7 min) and fixed with 4% formaldehyde (Invitrogen^®^, Grand Island, NY, USA) for 30–60 min at 4 °C. After centrifugation, the supernatant was discarded, and ice-cold 70% ethanol was added to the samples, which were stored at −20 °C until further analysis. On the day of analysis, the stored samples were thawed at room temperature, and the concentration of spermatozoa was adjusted to 2–3 × 10^6^ spermatozoa/mL with 70% ethanol. Positive and negative controls included in the APO-DIRECT™ Kit were additionally prepared. The examined and control samples were centrifuged at 1400 rpm for 7 min at room temperature. The supernatant was discarded, and the pellets were resuspended in 1 mL of wash buffer. After discarding the supernatant, 50 µL of staining solution was added to each sample. For each sample, the staining solution consisted of 0.75 μL of terminal deoxynucleotidyl transferase (TdT) enzyme, 8 μL of fluorescein isothiocyanate-2′-deoxyuridine-5-triphosphate (FITC-dUTP), 10 µL of reaction buffer, and 32.25 μL of distilled water. The negative control was prepared separately because it did not contain the TdT enzyme. All the samples were mixed and incubated for 60 min at 37 °C. Due to the photosensitivity of the enzyme, the samples were protected from light with aluminum foil. Following incubation, 1 mL of rinse buffer was added directly to the samples, which were subsequently centrifuged at 1400 rpm for 7 min. The supernatant was discarded, and the rinsing step was repeated. After removal of the supernatant, 0.5 mL of propidium iodide/RNase staining buffer was added to the samples, which were then incubated at room temperature for 30 min in the dark. Then, the samples were analyzed by flow cytometry within 3 h after staining using a MACSQuant Analyzer 10 flow cytometer with MACSQuantify 2.13.0 software (Miltenyi, Bergisch Gladbach, Germany). Two dyes were used: propidium iodide for total DNA staining and fluorescein isothiocyanate-2′-deoxyuridine-5-triphosphate for fragmented DNA staining. Based on the protocol described by Sharma et al. [106], negative control cells from the APO-DIRECT™ Kit were used to set the threshold to differentiate between fragmented and non-fragmented DNA. The results are expressed as the percentage of sperm with DNA fragmentation. A minimum of 10,000 events were recorded.

### 4.9. miRNA Analysis

#### 4.9.1. Spermatozoa Preparation

Semen samples from 30 men with normozoospermia and 20 men with oligozoospermia were used for miRNA expression analysis. The native samples and samples subjected to slow freezing or vitrification were washed two times with 1× PBS, and the pellets were stored at −80 °C until RNA isolation was performed.

#### 4.9.2. RNA Isolation

Total RNA was isolated from pelleted spermatozoa (if possible, at least 20 × 10^6^ spermatozoa from normozoospermic patients and at least 10 × 10^6^ spermatozoa from oligozoospermic patients) with a miRNeasy Mini Kit (217004, Qiagen, Hilden, Germany) according to the manufacturer’s protocol, with a modified sample lysis step described previously [81]. The yield and purity of the isolated RNA were assessed with a NanoDrop^TM^ One spectrophotometer and with a Qubit^TM^ RNA HS Assay Kit (Q32855) on a Qubit^TM^ 3.0 fluorometer (all Thermo Fisher Scientific, Waltham, MA, USA). The isolated total RNA was stored at –70 °C.

#### 4.9.3. Reverse Transcription

Reverse transcription was performed with the miRCURY LNA RT Kit (339340, Qiagen, Germany) in 10 μL reaction volumes. Briefly, each reaction mixture contained 2 μL of 5× miRCURY RT Reaction Buffer, RNase-free water, 1 μL of 10× miRCURY RT Enzyme Mix, 0.5 μL of UniSp6 RNA spike-in, and 10 ng (1 ng/μL) of total RNA template.

#### 4.9.4. Quantitative Real-Time PCR

Quantitative real-time PCR (qPCR) was performed in 10 µL reaction mixtures on a QuantStudio^TM^ 7 Pro Real-Time PCR System (Thermo Fisher Scientific, Waltham, MA, USA). All qPCR analyses were performed with a miRCURY SYBR Green PCR Kit (339347, Qiagen, Germany) and miRCURY LNA miRNA PCR Assays (339306; Qiagen, Germany) in accordance with the Minimum Information for Publication of Quantitative Real-Time PCR Experiments (MIQE) guidelines [107]. All qPCR reactions were performed in duplicate, and each 10 μL qPCR reaction mixture contained 5 μL of 2× miRCURY SYBR Green Master Mix, 1 μL of PCR primer mix, 0.05 μL of ROX Reference Dye, 0.95 μL of RNase-free water and 3 μL (0.05 ng) of cDNA template. The SNORD38B, SNORD44, and SNORD49A reference primers were used as endogenous controls for data normalization, and the UniSp6 primer was used as a reverse transcription positive control and as an inter-plate calibrator. The miRNA fold change was calculated by the 2^−ΔΔCt^ method [108], employing primer efficiency-corrected quantification cycle (Cq) values. Detailed information on the data normalization, Cq efficiency correction, and calculation of relative miRNA expression is available elsewhere [81]. Included miRNA primer assays are listed in Table 4.

### 4.10. Statistical Analysis

Statistical analysis was performed with IBM SPSS Statistics 27.0 software (IBM Corporation, Chicago, IL, USA). The normality of the data distribution was assessed by using Q–Q plots and the Kolmogorov-Smirnov and Shapiro-Wilk tests. Based on this evaluation, sperm concentration and sperm count were statistically evaluated by using the Mann-Whitney U test, and data obtained by the hyaluronan binding assay were evaluated by using Friedman’s two-way analysis of variance by ranks. For related samples, sperm motility was evaluated by Friedman’s two-way analysis of variance by ranks or by the Wilcoxon signed-rank test. As appropriate, sperm viability was evaluated by using either paired or unpaired Student’s *t* tests, and the results of protamine staining were evaluated by using ANOVA. miRNA expression in spermatozoa from normozoospermic individuals under different cryopreservation conditions was evaluated in a pairwise manner using paired Student’s *t* tests. To test the difference between miRNA expression in spermatozoa from patients with oligozoospermia under different sperm cryopreservation conditions, an unpaired Student’s *t* test was used. When comparing miRNA expression between spermatozoa from patients with oligozoospermia and normal spermatozoa, under each sperm cryopreservation condition, we evaluated the data by using the unpaired Student’s *t* test. In cases where data was non-normally distributed, the Mann-Whitney U test was used. Equality of variance was assessed by Levene’s test. All tests were two-tailed, and a *p* value < 0.05 was considered to indicate statistical significance.

## Figures and Tables

**Table 1:** The results of semen quality parameter assessment and sperm functional tests in native normozoospermic samples and comparison to slow-frozen and vitrified paired samples. A *p* value of <0.05 was considered to indicate statistical significance.

	Native Semen	Slow Freezing of Semen	Vitrification of Semen	*p* Value
Total motility (%)	60.0 (50.0–70.0)	18.9 (11.9–24.3)	13.8 (7.9–20.9)	<0.001
Fast progressive motility (%)	45.0 (33.8–50.0)	7.5 (3.4–11.8)	5.4 (1.7–9.3)	<0.001
Slow progressive motility (%)	10.0 (10.0–10.0)	6.0 (3.5–8.6)	4.3 (2.5–9.5)	<0.001
Non-progressive motility (%)	5.0 (5.0–10.0)	3.2 (2.1–5.3)	2.7 (1.4–4.4)	<0.001
Viability (mean ± SD)	/	19.7 ± 11.4	21.3 ± 11.9	0.346
Protamine staining (mean ± SD)	53.6 ± 15.1	53.9 ± 16.0	52.4 ± 15.2	0.905
Sperm DNA fragmentation (mean ± SD)	18.7 ± 9.5	24.7 ± 11.6	27.1 ± 15.7	0.049
Mature spermatozoa according to HBA assay	78.0 (67.5–82.4)	79.4 (71.7–82.9)	75.3 (67.5–81.1)	0.607

Values are reported as medians with interquartile ranges (Q1–Q3), except for viability, protamine staining, and sperm DNA fragmentation, which are reported as the means with standard deviations.

**Table 2:** The results of semen quality parameter assessment in native oligozoospermic samples and comparison to those of slow-frozen and vitrified samples. A *p* value of < 0.05 was considered to indicate statistical significance.

	Native Semen before Slow Freezing	Semen after Slow Freezing	*p* Value	Native Semen before Vitrification	Semen after Vitrification	*p* Value
Sperm concentration (×10^6^/mL)	11 (6.5–12.5)	/		9.5 (7–12)	/	0.976
Total sperm count (×10^6^)	45 (27–58.5)	/		44.9 (31.7–50.5)	/	0.741
Total motility (%)	45 (25–55)	9.4 (5.8–20.0)	<0.001	40 (30–47.5)	7.9 (6.1–15.2)	<0.001
Fast progressive motility (%)	30 (12.5–37.5)	3 (0.4–4.1)	<0.001	25 (17.5–30)	2.4 (0–4.9)	0.002
Slow progressive motility (%)	10 (5–12.5)	3.6 (2.2–6.4)	0.003	10 (7.5–10)	2.5 (0.9–3.3)	<0.001
Non-progressive motility (%)	5 (5–5)	2.7 (1.6–5.8)	0.016	5 (5–5)	3.7 (1.5–5.3)	0.112
Viability (mean ± SD)		15.1 ± 7.7			12.4 ± 6.5	

Values are reported as medians with interquartile ranges (Q1–Q3), except for viability, which is reported as the mean value with standard deviation.

**Table 3:** The list of miRNAs analyzed in our study and their interrelation with sperm quality in humans.

miRNA	miRNA Expression ^a^	Reference
let-7a-5p	↓ in men with teratozoospermia.	[81]
	↑ in spermatogenic failure.	[88]
	↑ in infertile men—negative correlation between let-7a expression and sperm concentration.	[65]
	↑ in men with non-obstructive azoospermia.	[91]
	↓ in infertile normozoospermic and asthenozoospermic men. Positive correlation with motility.	[89]
miR-10a-5p	↓ in men with teratozoospermia.	[81]
	↑ in men with non-obstructive azoospermia.	[92]
	↑ miR-10a in men with non-obstructive azoospermia with meiotic arrest.	[43]
	↓ in testes of Klinefelter syndrome patients.	[93]
miR-15b-5p	Positive correlation between miR-15b expression and sperm concentration.	[65]
	↓ in men with oligoasthenozoospermia.	[94]
	↓ in men with oligozoospermia.	[64]
	↑ in infertile normozoospermic men.	[80]
	↑ in men with non-obstructive azoospermia.	[95]
	↓ in men with teratozoospermia.	[81]
	↓ in testes of Klinefelter syndrome patients.	[93]
miR-26a-5p	↓ in unexplained infertile men—correlation with motility and normal morphology.	[90]
	↓ in infertile men with semen abnormalities.	[96]
	↓ in men with teratozoospermia.	[81]
	↑ in men with oligoasthenozoospermia and asthenozoospermia.	[94]
miR-34b-3p	Biomarker potential of miR-34b-3p and miR-93-3p for unexplained male infertility.	[63]
	↓ in non-obstructive and obstructive azoospermia patients, and Klinefelter syndrome patients.	[97]
	↑ associated with normal spermatogenesis and the potential of retrieving spermatozoa during testicular biopsy.	[98]
	miR-34b expression significantly associated with ICSI outcomes in male infertility (teratozoospermia).	[66]
	Positive correlation between miR-34b expression and sperm concentration.	[65]
	↓ in men with teratozoospermia.	[81]
	Differential expression of miR-34b-3p in men with oligozoospermia and asthenozoospermia.	[64]
	↓ in men with oligoasthenozoospermia and asthenozoospermia.	[94]
miR-92a-3p	↓ in the high blastocyst rate group in idiopathic infertile men.	[99]
	↑ in men with asthenozoospermia.	[94]
	↓ in testes of Klinefelter syndrome patients.	[93]
	↓ in testes with Sertoli cell-only syndrome.	[100]
miR-93-3p	Biomarker potential of miR-93-3p and miR-34b-3p for unexplained male infertility.	[63]
	↑ in infertile normozoospermic men.	[80]
miR-99b-5p	miR-99b-5p expression in spermatozoapositively correlated with good-quality day 3 embryos.	[81]
	↑ in men with asthenozoospermia.	[94]
	↑ in testes with Sertoli cell-only syndrome and germ cell arrest.	[100]
miR-122-5p	↓ in men with oligozoospermia—positive correlation with sperm density.	[101]
	↓ in men with non-obstructive and obstructive azoospermia, and men with Klinefelter syndrome.	[97]
	↓ in men with oligo/oligoasthenozoospermia, infertile men with normozoospermia, and men with asthenozoospermia—positive correlation with sperm concentration and motility.	[89]
	↑ in infertile males with semen abnormalities.	[96]
	Positive correlation between miR-122 expression and sperm concentration.	[65]
	↑ in men with asthenozoospermia.	[66]
	↓ in men with teratozoospermia.	[81]
	↓ of seminal miR-122 in men with oligoasthenozoospermia and varicocele.	[102]
miR-125b-5p	↓ in men with teratozoospermia.	[81]
	↑ in testes of Klinefelter syndrome patients.	[93]
	↓ in testes with Sertoli cell-only syndrome.	[100]
miR-191-5p	Sperm miR-191-5p expression associated with early human embryonic quality.	[103]
	↓ in men with teratozoospermia.	[81]
miR-296-5p	Biomarker potential of miR-296-5p and miR-328-3p in men with teratozoospermia.	[63]
	↓ in men with teratozoospermia.	[81]
	↑ in infertile normozoospermic men.	[80]
	↓ in smokers’ spermatozoa.	[104]

^a^ miRNA expression and its role in the context of sperm quality in humans. The arrows represent miRNA overexpression (↑) or under-expression (↓), compared to the controls.

**Table 4:** miRNA primer assays.

miRNA	Catalog Number ^a^
let-7a-5p	YP00205727
miR-10a-5p	YP00204778
miR-15b-5p	YP00204243
miR-26a-5p	YP00206023
miR-34b-3p	YP00204005
miR-92a-3p	YP00204258
miR-93-3p	YP00204470
miR-99b-5p	YP00205983
miR-122-5p	YP00205664
miR-125b-5p	YP00205713
miR-191-5p	YP00204306
miR-296-5p	YP00204436
SNORD38B ^b^	YP00203901
SNORD44 ^b^	YP00203902
SNORD49A ^b^	YP00203904
UniSp6 ^c^	YP00203954

^a^ miRCURY LNA miRNA PCR Assay catalog number (product number 339306; Qiagen, Germany). ^b^ Reference miRNA PCR assays used for data normalization. ^c^ miRNA PCR assay specific for amplification of UniSp6 RNA spike-in control.

## Data Availability

The raw data supporting the conclusions of this article will be made available by the authors upon request.
